# Seasonal Changes in Vitamin D-Effective UVB Availability in Europe and Associations with Population Serum 25-Hydroxyvitamin D

**DOI:** 10.3390/nu8090533

**Published:** 2016-08-30

**Authors:** Colette M. O’Neill, Andreas Kazantzidis, Mary J. Ryan, Niamh Barber, Christopher T. Sempos, Ramon A. Durazo-Arvizu, Rolf Jorde, Guri Grimnes, Gudny Eiriksdottir, Vilmundur Gudnason, Mary Frances Cotch, Mairead Kiely, Ann R. Webb, Kevin D. Cashman

**Affiliations:** 1 Cork Centre for Vitamin D and Nutrition Research, School of Food and Nutritional Sciences, University College Cork, Cork T12 YN60, Ireland; colette.oneill@ucc.ie (C.M.O.); 112361636@umail.ucc.ie (M.J.R.); 112473748@umail.ucc.ie (N.B.); m.kiely@ucc.ie (M.K.); 2 Laboratory of Atmospheric Physics, Physics Department, University of Patras, Patras 26504, Greece; akaza@upatras.gr; 3 School of Earth Atmospheric and Environmental Sciences, University of Manchester, Manchester M13 9PL, UK; ann.webb@manchester.ac.uk; 4 Office of Dietary Supplements, National Institutes of Health, Bethesda, Montgomery County, MD 20892, USA; semposch@od.nih.gov; 5 Department of Public Health Sciences, Loyola University Stritch School of Medicine, Chicago, IL 60153, USA; rdurazo@luc.edu; 6 Tromsø Endocrine Research Group, Department of Clinical Medicine, UiT The Arctic University of Norway, Tromsø 9019, Norway; Rolf.Jorde@unn.no (R.J.); Guri.Grimnes@unn.no (G.G.); 7 Icelandic Heart Association, Kopavogur IS-201, Iceland; gudny@hjarta.is (G.E.); villi@hjarta.is (V.G.); 8 University of Iceland, Reykjavik IS-101, Iceland; 9 Division of Epidemiology and Clinical Applications, National Eye Institute, National Institutes of Health, Bethesda, Montgomery County, MD 20892, USA; mfc@nei.nih.gov; 10 Irish Centre for Fetal and Neonatal Translational Research, University College Cork, Cork T12 YN60, Ireland; 11 Department of Medicine, University College Cork, Cork T12 YN60, Ireland

**Keywords:** vitamin D status, UVB, Europe, vitamin D intake

## Abstract

Low vitamin D status is common in Europe. The major source of vitamin D in humans is ultraviolet B (UVB)-induced dermal synthesis of cholecalciferol, whereas food sources are believed to play a lesser role. Our objectives were to assess UVB availability (Jm^−2^) across several European locations ranging from 35° N to 69° N, and compare these UVB data with representative population serum 25-hydroxyvitamin D (25(OH)D) data from Ireland (51–54° N), Iceland (64° N) and Norway (69° N), as exemplars. Vitamin D-effective UVB availability was modelled for nine European countries/regions using a validated UV irradiance model. Standardized serum 25(OH)D data was accessed from the EC-funded ODIN project. The results showed that UVB availability decreased with increasing latitude (from 35° N to 69° N), while all locations exhibited significant seasonal variation in UVB. The UVB data suggested that the duration of vitamin D winters ranged from none (at 35° N) to eight months (at 69° N). The large seasonal fluctuations in serum 25(OH)D in Irish adults was much dampened in Norwegian and Icelandic adults, despite considerably lower UVB availability at these northern latitudes but with much higher vitamin D intakes. In conclusion, increasing the vitamin D intake can ameliorate the impact of low UVB availability on serum 25(OH)D status in Europe.

## 1. Introduction

Vitamin D deficiency has significant implications for human health throughout the lifecycle [1]. Recent data on the prevalence of vitamin D deficiency in Europe shows that, on average, one in eight individuals have a serum 25-hydroxyvitamin D (25(OH)D) concentration < 30 nmol/L, reflective of vitamin D deficiency [2]. The major source of vitamin D in humans is the ultraviolet B (UVB) radiation in sunshine, with estimates of cutaneous synthesis providing 80%–100% of the vitamin D requirements of the body [3]. Vitamin D_3_ synthesis begins with UVB radiation reaching 7-dehydrocholesterol in the skin, and there are a number of personal and environmental factors that can help or hinder this first step [4]. The personal attributes include skin pigmentation, age, attire, sunscreen usage, working environment, outdoor physical activity, and sun exposure behavior, while the environmental factors include season, latitude, time of day, prevailing weather conditions, ozone, amongst others [1,4]. Key environmental factors such as season, latitude, and time of day, which determine if UVB radiation of sufficient strength is available to potentially stimulate cutaneous synthesis of pre-vitamin D_3_, are associated with changing solar zenith angle (SZA) [3,4].

The SZA is the angle between the local vertical [zenith] and the position of the sun in the sky at any given moment [4]. When the SZA is large the incoming radiation at the top of the atmosphere is spread over a large area, then the UVB radiation has to travel a long slanting path in the atmosphere, where it undergoes attenuation. At UV wavelengths attenuation is mainly from UV-absorbing ozone and Rayleigh scattering, whose effect depends on the inverse fourth power of the wavelength and so is particularly strong at UV wavelengths (it is this effect that makes the sky look blue as blue light is scattered across the sky hemisphere much more effectively than red light). The net effect at large SZA is very small amounts of UVB reaching the ground; whereas when the SZA is small, incoming radiation is concentrated over a smaller area, UVB has a relatively short path-length through the atmosphere (less attenuation) and yields greater amounts reaching the ground [3,4]. As an example, small SZAs are associated with noon, summer, and low latitudes, whereas large SZAs are a feature of early morning/late afternoon, and winter, particularly at high latitudes, but also high latitudes per se [4], associations mirrored by UVB availability. Thus, it is not surprising to see that when prevalence of vitamin D deficiency in our pan-European assessment was stratified by whether subject sampling was in extended winter or summer (November to March, and April to October, respectively), the prevalence of serum 25(OH)D < 30 nmol/L was 17.7% and 8.3%, respectively [2]. 

While Europe spans from ~35° N to ~70° N, one might assume differences in UVB availability, based on changing SZAs, would be mirrored by differences in prevalence of vitamin D deficiency but this is not always the case. In adults and older adults, the UVB-limited Northern European countries have been shown to have lower prevalence of vitamin D deficiency than that in the mid-latitude European countries [2,5,6], but in childhood population samples, the countries of Central and Northern Europe (47–60° N) have been shown to have a higher prevalence range (5%–20%) than that in the southern countries (<41° N) at 4%–7% [2]. 

We wished to map the UVB availability across European locations and examine whether, together with some behavioral aspects, it may further inform some of our vitamin D status findings for Europe. To achieve this, the present work used UV dose data generated from a validated UV irradiance model and applied to a selection of European countries/regions which formed the core of the recent pan-European assessment of prevalence of vitamin D deficiency [2]. These locations spread from relatively southerly (Crete, Greece; ~35° N) to northerly (Tromsø, Norway; 69° N) parts of Europe, providing good latitudinal coverage. In addition, we used standardized serum 25(OH)D data, which avoids methods-related differences in estimates [7], from a selection of these European countries/regions to explore the potential impact of UVB availability on their respective population vitamin D status. 

## 2. Materials and Methods

### 2.1. Selection of European Sites to Model UVB Availability

The European Commission-funded “Food-based solutions for optimal vitamin D nutrition and health through the life cycle” (*ODIN*; www.odin-vitd.eu) project and an associated Norden-funded small project had as one of their key overall objectives to quantify the prevalence of vitamin D deficiency in European populations using standardized serum 25(OH)D values [2,8]. This was only feasible by the standardization of serum 25(OH)D data via the NIH-led international Vitamin D Standardization Program (VDSP) and its protocols for standardizing existing 25(OH)D values from national health/nutrition surveys [7]. The projects included a number of identifiable nationally representative nutrition and health surveys in addition to regionally representative health surveys from various European member states and of different life stage groups, which were of strategic importance for European coverage. Cashman and colleagues recently applied these VDSP protocols to serum 25(OH)D data from these 18 representative childhood/teenage and adult/older adult European populations, to better quantify the prevalence of vitamin D deficiency in Europe [2,8]. The countries and/or regions represented in this pan-European assessment of prevalence of vitamin D deficiency, namely Ireland, UK, Germany, Finland, Denmark (Aarhus and Copenhagen), Greece (Thessaloniki, Athens, and Crete), Norway (Oslo and Tromsø), Netherlands (Amsterdam and Hoorn), and Iceland (Reykjavik), were again selected as exemplars in the present analysis as they represent a sizable geographical footprint for which to assess patterns of UVB availability in Europe. 

### 2.2. Generation of UV Dose Data for European Sites

The present work used UV dose data generated from a UV irradiance model also implemented within the ODIN project. Full details of this model have been presented elsewhere [9], but in brief, the ambient UV irradiance on a 1° × 1° degree latitude/longitude grid at the above mentioned countries/regions (see Table 1 for coordinates used) were generated using the UVSPEC radiative transfer model together [10] with inputs of local cloud, ozone, and aerosol, plus topography at a temporal resolution of 15 min for a period of 10 years (2003–2012 period). Data on these atmospheric and geophysical parameters are required for the calculation of UV irradiance at the ground. As input data was required across Europe, the only feasible method of acquiring such data with the resolution required was to use satellite products, as follows in brief: the cloud optical depth and fraction values were derived from the MODerate resolution Imaging Spectroradiometer (MODIS) instrument (http://modis.gsfc.nasa.gov), on board the Terra and Aqua satellites. Total ozone data was acquired from the Total Ozone Mapping Spectrometer (TOMS) on board the Earth Probe satellite and the Ozone Monitoring Instrument (OMI) on board the Aura satellite. Aerosol Optical Depth (AOD) values at 550 nm from the MODIS Terra and Aqua daily (Level-3 data) were used to build the aerosol climatology across Europe. The Air-Force Geophysical Laboratory (AFGL) standard profiles of temperature, air-pressure, ozone, and the basic atmospheric gases over mid-latitudes for winter (considered appropriate for the October–March period) and summer (April–September) were also used as model inputs. A converted version of GTOPO30 digital elevation model was used to account for altitude. The TEMIS website [11] provides converted altitude data at different resolution, in this study, the 1° × 1° resolution was used. Monthly climatological values of the 340–380 nm Lambertian equivalent surface reflectivity from TOMS instrument onboard Nimbus-7 satellite were used. 

This model has been validated against ground-based measurements from a number of sites across the UK and Ireland [9,12]. Modeled spectra were weighted with the Commission Internationale de l’Eclairage (CIE) action spectrum for the production of pre-vitamin D_3_ in human skin [13]. There are some uncertainties in the CIE action spectrum due to the data on which it is based (fully discussed in the original document [13]). Various attempts to better define the action spectrum have been made [14] but none have succeeded in a more robust definition than that of CIE, and have concluded that until new data are available it is better to maintain use of a common action spectrum (as defined by CIE). Data are provided in Jm^−2^ for each location. The modelled UVB dose data for each location over the 10 year period was averaged into daily and, subsequently, monthly estimates and in light of the uncertainties of the values that go into the model calculations we have rounded to the nearest 100 Jm^−2^. In the present work, a threshold of 1000 Jm^−2^ was used as a guide to a UVB dose below which dermal synthesis of pre-vitamin D_3_ will be relatively low. Of importance, it should not be viewed as an empirical UVB dose below which dermal synthesis of pre-vitamin D_3_ is comprised, but rather reflects a dose which is only likely to lead to minimal increases in average monthly serum 25(OH)D (i.e., <1.5 nmol/L) during winter. This was based on our previous modeling of the direct impact of UVB on population serum 25(OH) D [12]. It is also in line with the older data from Webb et al. [15] who, using human skin or [3α-^3^H]7-dehydrocholesterol in a model system exposed to sunlight, showed that pre-vitamin D_3_ was not produced for 4 and 6 months of winter at 42° N and 52° N, respectively. The number of months in the typical year (mean of 10 years) for which the mean modeled UV dose < 1000 Jm^−2^ was used as an estimate of the duration of the “vitamin D winter” in each location. In addition, the percentage of days in the typical year that had mean modeled UV dose < 1000 Jm^−2^ was also calculated for each location.

### 2.3. Estimating the Cosine Solar Zenith Angle for European Sites

The cosine of SZA at each location was estimated using the online US National Oceanic and Atmospheric Administration’s (NOAA) Earth System Research Lab (ESRL) Solar Position Calculator [16]. The cosine of SZA was estimated at true solar noon on 21 June (typical time of the summer solstice) in 2008, as an approximate mid-point of the 10 year period (2003–2012) used to model UVB doses. 

### 2.4. Standardized Serum 25-Hydroxyvitamin D Concentration for Four Selected Population Samples

Standardized serum 25(OH)D data from three of the eight adult/older adult study populations and one of the six childhood/adolescent study populations within the ODIN project were used in the present work to illustrate seasonal variations in UV availability and associated population vitamin D status. We used regionally/nationally representative data from Tromsø, Norway (Tromsø six adult cohort and Tromsø Study: Fit Futures (an adolescent population study); 69° N) [17,18], Reykjavik, Iceland (AGES-Reykjavik cohort; 64° N) [19] and Ireland (National Adult Nutrition Survey; 51–54° N) [20]. The VDSP protocol for standardization of serum 25(OH) D data from past surveys has been described in detail elsewhere [7], and specific details of its application to these 4 population samples has been presented recently [2,21]. The liquid chromatography-tandem mass spectrometry (LC-MS/MS) method at the Cork Centre for Vitamin D and Nutrition Research, which is certified by the Centers for Disease Control and Prevention’s Vitamin D Standardization Certification Program [22], was used for all four population samples included in this work. The Cork Centre for Vitamin D and Nutrition Research is a participant in the VDSP [7] and, in addition, the quality and accuracy of the serum 25(OH)D analysis by using the LC-MS/MS in our laboratory is monitored on an on-going basis by participation in the Vitamin D External Quality Assessment Scheme (Charing Cross Hospital, London, UK). The mean and standard deviation of serum 25(OH)D concentrations were calculated for each available month within these four populations. 

### 2.5. Statistical Analysis

Differences between mean monthly UVB doses over a typical year between countries within a latitude band were evaluated by pair-wise comparisons. *p* ≤ 0.05 were taken as significant.

## 3. Results

**Figure 1:**
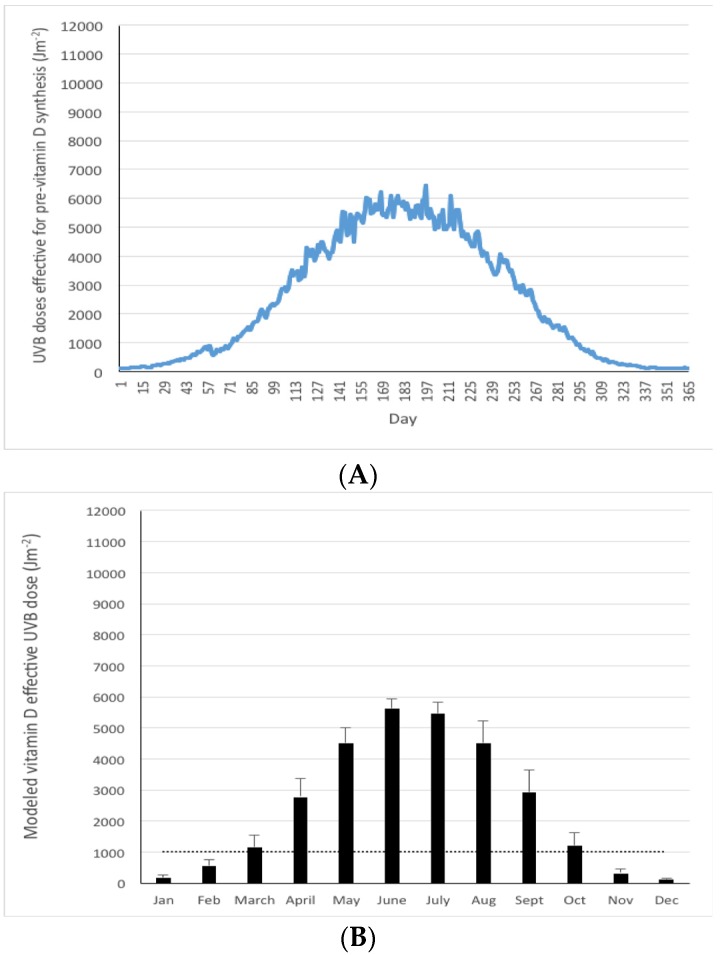
Mean modeled UVB doses effective for pre-vitamin D_3_ synthesis (Jm^−2^) in Germany (47–55° N) on a daily (**A**) and monthly (**B**) basis in a typical year (mean of 2003–2012). Dotted line in panel B reflects a threshold of 1000 Jm^−2^ as a guide to a dose below which dermal synthesis of pre-vitamin D_3_ is relatively low.

The modeled vitamin D effective UVB doses (“modeled UVB doses” henceforth) on a daily basis throughout a typical year (mean of 10 years) for Germany, as an exemplar, are shown in Figure 1a. As might be expected, there was significant variation in UVB dose from day to day as the atmosphere (weather) changed, particularly during the summer period when doses where at the highest. For example, in July daily modeled UVB dose ranged from a minimum of 4900 Jm^−2^ to a maximum of 6400 Jm^−2^ (data not shown). Using a threshold of 1000 Jm^−2^ as that below which dermal synthesis of pre-vitamin D_3_ is relatively low, shows that it was only mid-March before UVB doses exceeded this threshold, increasing during summer months and decreasing to levels below this threshold by the latter part of October. A mean monthly modeled UVB dose (defined as the mean daily dose for the month) was also calculated from the modeled daily doses for each month (Figure 1B), which was a simpler representation of UVB dose fluctuations over the course of a typical year. Presentation of mean monthly modeled UVB doses was adopted for the various countries and regions in the remainder of the analysis. Mean monthly modeled UVB doses in December and January were extremely low (<200 Jm^−2^).

The duration of the “vitamin D winter” in Germany, based on the number of months in the typical year for which the mean monthly modeled UVB doses < 1000 Jm^−2^ was 4 (Figure 1b and Table 1). Over a third of the year (40%) had mean modeled daily UVB doses < 1000 Jm^−2^. Data on the 3 composite 1° × 1° grids, used to provide coverage of Germany (latitude bands 47.4–49.9, 50.0–51.9, and 52.0–54.4° N, respectively), and from which a mean for Germany was calculated from, shows there was a trend for mean monthly modeled UVB doses in each of the 12 months to decrease with increasing latitude (data not shown). Similarly, yearly mean modeled UVB doses decreased with increasing latitudinal band (*p* < 0.0001).

**Figure 2:**
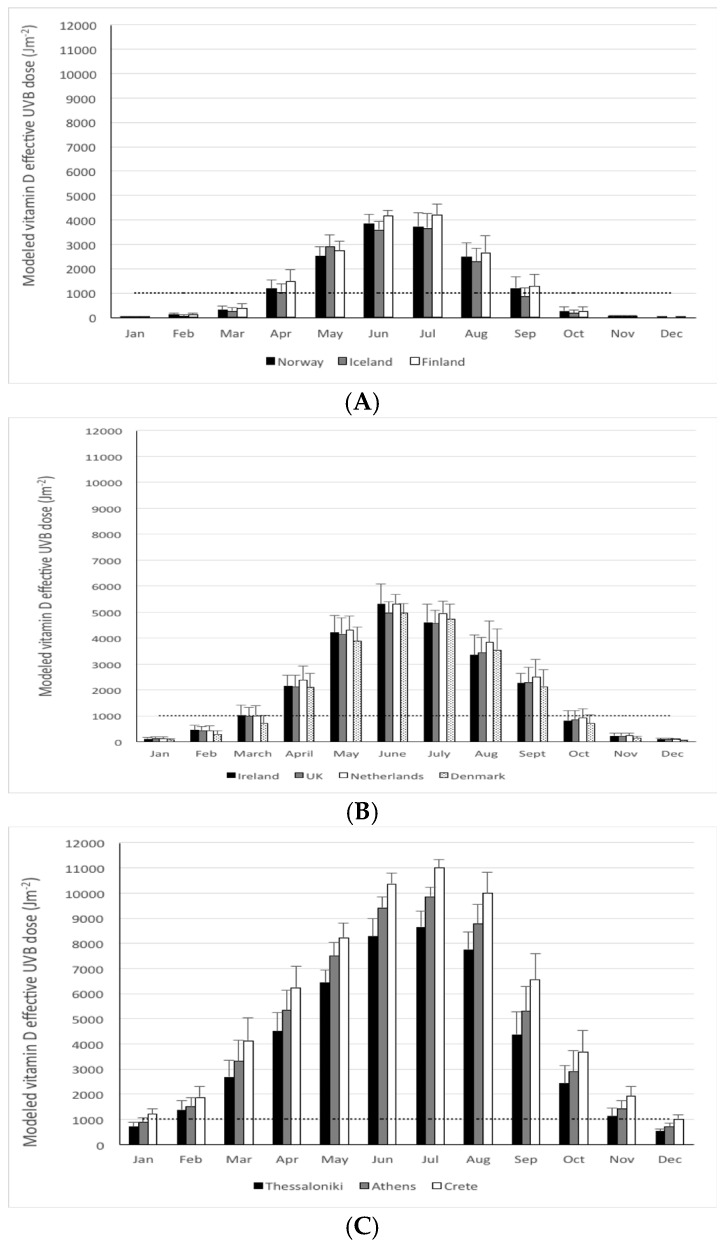
Mean modeled UVB doses effective for pre-vitamin D_3_ synthesis (Jm^−2^) in Norway (mean of Oslo (60° N) and Tromsø (69° N)), Iceland (Reykjavik (64° N)) and Finland (60–70° N) (**A**); Ireland (51–54° N), the UK (50–59° N), Netherlands (Amsterdam, (52° N)), and Denmark (mean of Copenhagen and Aarhus (56° N)) (**B**); and Thessaloniki (40° N), Athens (37° N) and Crete (35° N) (**C**) on a monthly basis in a typical year (mean of 2003–2012). Dotted line reflects a threshold of 1000 Jm^−2^ as a guide to a dose below which dermal synthesis of pre-vitamin D_3_ is relatively low.

The mean monthly modeled UVB doses for Norway, Iceland, and Finland, all above 60° N, are shown in Figure 2A. Relative to that in Germany (spanning ~47–54° N), the monthly mean modeled UVB doses were lower in each of these three Northern European countries (Table 1). Maximum monthly mean modeled UVB doses was close to only 4000 Jm^−2^ in these three countries compared to 5600 Jm^−2^ in Germany. In general, Finland had slightly higher mean monthly modeled UVB doses than either Norway or Iceland, with only relatively small differences between these latter two countries (Figure 2A). This is also reflected in a significantly higher mean yearly modeled UVB dose for Finland than either Iceland or Norway (*p* < 0.02 in both cases), with no significant difference in these latter two countries (*p* = 0.2) (Table 1). November through to February, inclusive, had mean monthly modeled UVB doses < 100 Jm^−2^ in all three countries. The “vitamin D winter” lasted for six months in both Norway and Finland, and extended to a seventh month in Iceland (Figure 2 and Table 1). However, within Norway, Oslo (60° N) and Tromsø (69° N) had 51% and 64% of the year with mean daily modeled UVB doses < 1000 Jm^−2^, respectively, leading to “vitamin D winters” lasting for six and eight months, respectively. 

The mean monthly modeled UVB doses for Ireland, the UK, Netherlands, and Denmark, all in a latitude band of 50–60° N, are shown in Figure 2B. The maximum monthly mean modeled UVB doses was 5000 Jm^−2^ in these four countries (Figure 2B), a little lower than that in Germany, but higher than that of the three more Northern European countries. Taking the full year into account, the Netherlands may have had slightly higher mean monthly modeled UVB doses than the other three countries, while Denmark may have had slightly lower (Figure 2B). This is also reflected in a significantly higher mean yearly modeled UVB dose for the Netherlands than any of the other three countries (*p* < 0.03 in all cases), with no other significant differences between countries (*p* > 0.05) (Table 1). However, there was a trend for Denmark to have a lower mean yearly modeled UVB dose compared to Ireland (*p* = 0.059) and the UK (*p* = 0.083). November through to January, inclusive, had mean monthly modeled UVB doses < 300 Jm^−2^ in all four countries. The “vitamin D winter” lasted for five months in Ireland, the UK, and the Netherlands, and extended to a sixth month in Denmark (Figure 2B and Table 1). 

The mean monthly modeled UVB doses for three parts of Greece, all in a latitude band of 35–41° N, are shown in Figure 2C. Maximum monthly mean modeled UVB doses was 9800 Jm^−2^, on average, for these three Greek regions combined (Figure 2C), much higher than that in any of the other European countries in the present analysis. Within the region, the maximum monthly mean modeled UVB doses decreased with latitude (11,000, 9900, and 8600 Jm^−2^ for Crete (35.2° N), Athens (38.0° N) and Thessaloniki (40.6° N), respectively), and the clear latitudinal trend was evident in the mean monthly modeled UVB doses for each of the 12 months (Figure 2C) as well as in yearly mean (*p* < 0.0001) (Table 1). None of the three Greek regions had mean monthly modeled UVB doses < 500 Jm^−2^ at any stage. The “vitamin D winter” lasted for two months in Thessaloniki in Northern Greece and Athens, but was absent in Crete (Figure 2C and Table 1). 

**Figure 3:**
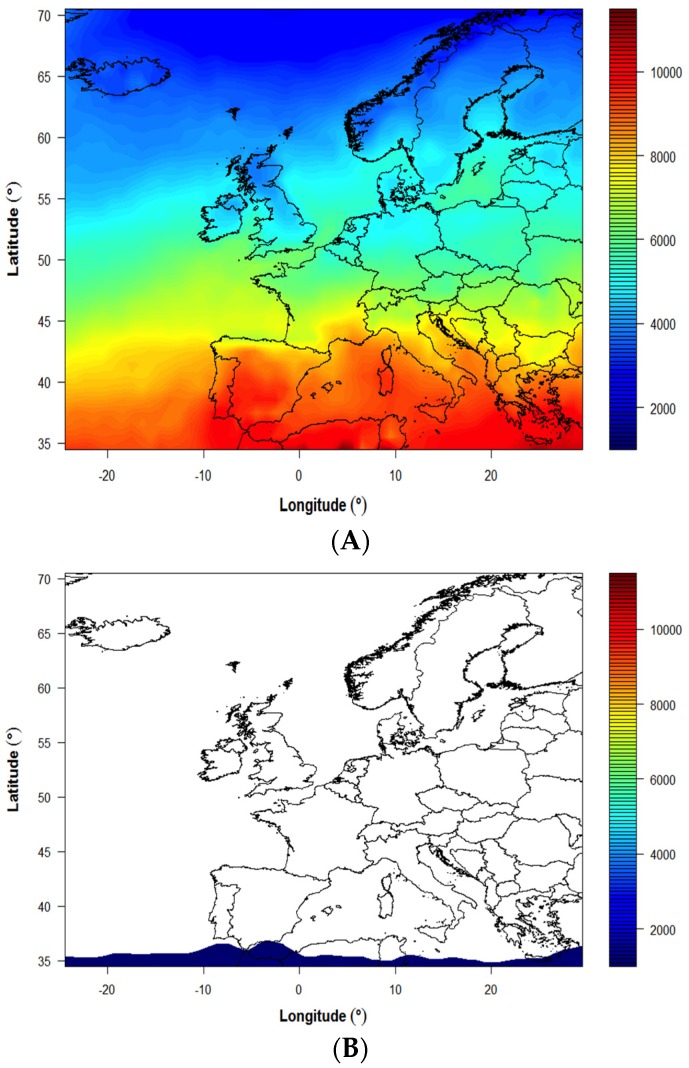
Mean monthly modeled UVB doses effective for pre-vitamin D_3_ synthesis (Jm^−2^) across Europe for June (**A**) and December (**B**), based on average of data from years 2003–2012. Scale begins 1000 Jm^−2^.

The variability in modeled UVB availability over Europe overall during both June and December is shown in Figure 3. The decreasing UVB availability with increasing latitude within the range ~35–70° N is very evident in summer (Figure 3A), and is associated with increasing SZA (as evidenced by the cosine of SZA at true solar noon on 21 June; Table 1). The lack of UVB dose > 1000 Jm^−2^ throughout all of Europe during December is also very striking (Figure 3B), while decreasing UVB availability (within the range 0–1000 Jm^−2^) with increasing latitude is still evident (data not shown).

**Figure 4:**
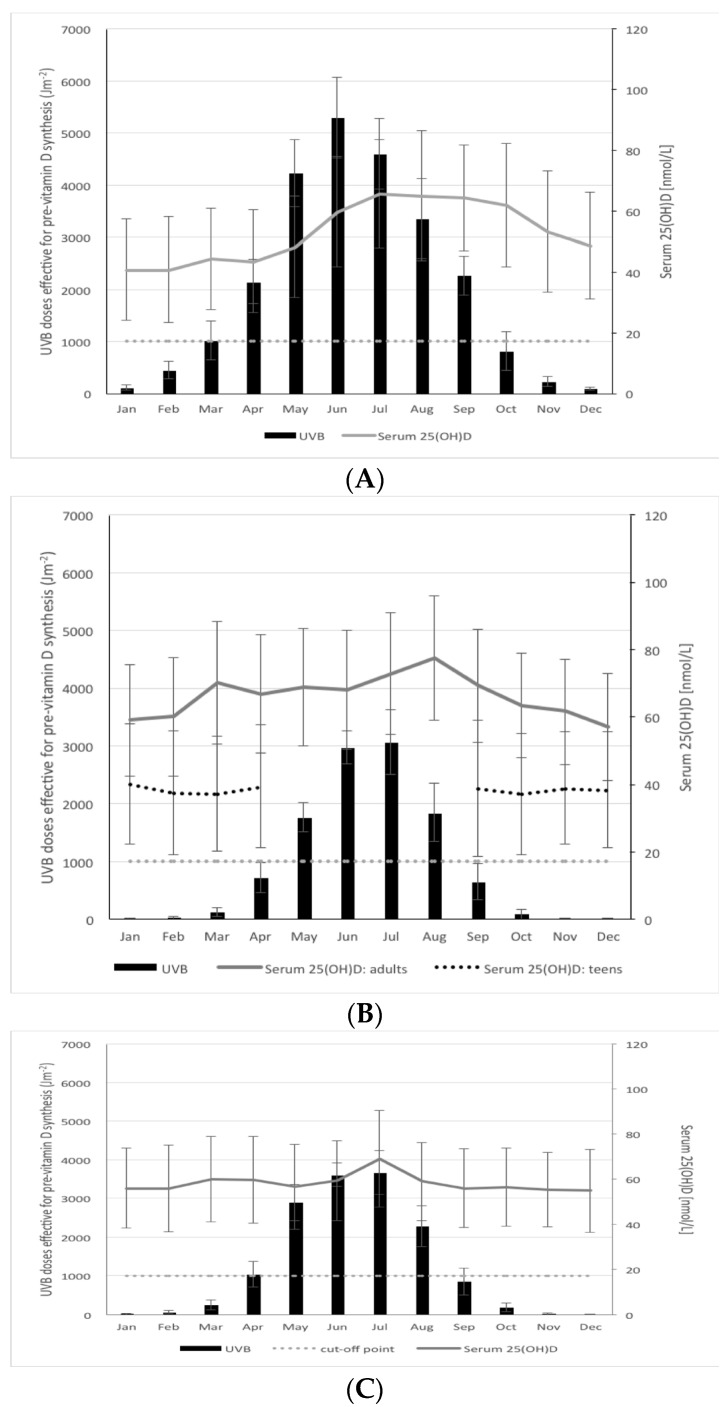
Mean modeled UVB doses effective for pre-vitamin D_3_ synthesis (Jm^−2^) on a monthly basis in a typical year (mean of 2003–2012) in Ireland (51–54° N) and mean (SD) monthly serum 25(OH)D measured in adults (18–84 years) in the National Adult Nutrition Survey in Ireland [21] (**A**); in Tromsø, Northern Norway (69° N) and mean (SD) monthly serum 25(OH)D measured in adults (18–83 years) in the regionally representative Tromsø sixth cycle cohort [17] (solid line) and adolescents (15–18 years) in the Tromsø—Fit Futures study [18] (dotted black line) (**B**); and in Reykjavik, Iceland (64° N) and mean (SD) monthly serum 25(OH)D measured in adults (aged 66–96 years) in the regionally representative AGES-Reykjavik cohort in Iceland [19] (**C**). Note: Data on serum 25(OH)D for the four summer months (May to August) are absent in Tromsø—Fit Futures study as the adolescents were not sampled in this period due to school summer vacation. Dotted line reflects a threshold of 1000 Jm^−2^ as a guide to a dose below which dermal synthesis of pre-vitamin D_3_ is relatively low. Black bars and error bars represent mean and SD monthly UVB doses, respectively.

The mean (and SD) for the monthly standardized serum 25(OH)D values in the Irish adults (aged 18–84 years) from the nationally representative NANS [20] have been superimposed onto the graph of monthly mean modeled UVB doses in Ireland (51–54° N) (Figure 4A). There was a clear seasonal variation in serum 25(OH)D concentration of Irish adults (*p* < 0.0001) with a late summer peak and late winter nadir, separated by ~25 nmol/L. The seasonal fluctuations in serum 25(OH)D concentrations broadly tracks, albeit with a slight lag, that of the UVB availability for Ireland. 

The mean (and SD) for the monthly standardized serum 25(OH)D values in the adults (aged 30–87 years) from the sixth cycle of the regionally representative Tromsø cohort [17] have been superimposed onto the graph of monthly mean modeled UVB doses in Tromsø, Northern Norway (69° N) (Figure 4B). Likewise, the mean (and SD) for the monthly standardized serum 25(OH)D values in the older adults (aged 66–96 years) from the regionally representative AGES-Reykjavik cohort [19] have been superimposed onto the graph of monthly mean modeled UVB doses in Reykjavik, Iceland (64° N) (Figure 4C). While there was seasonal variation in serum 25(OH)D concentration of adults in Reykjavik and Tromsø (*p* < 0.0001 in both cases), it was more blunted than that seen in the Irish adults, with ~14 nmol/L and ~20 nmol/L from peak to nadir differences, respectively. The winter nadirs in serum 25(OH)D concentration in both of these Northern European populations (54.8 and 56.9 nmol/L, respectively), were much higher than that of the Irish adult population (40.6 nmol/L), despite much reduced UVB availability in the former countries and “vitamin D winters” of seven and eight months for Reykjavik and Tromsø, respectively. Even in the height of respective summers in these countries, peak mean serum 25(OH)D concentrations were 65.7, 68.9, and 77.4 nmol/L for the adults in Ireland. 

Reykjavik and Tromsø, respectively, and despite mean monthly modeled UVB doses (for June and July) of 4900, 3600 and 3000 Jm^−2^, for these 3 regions, respectively.

The mean (and SD) for the monthly standardized serum 25(OH)D values in the adolescents (aged 15–18 years) from the Tromsø Fit Futures study [18] have also been superimposed onto the graph of monthly mean modeled UVB doses in Tromsø, Northern Norway (69° N) (Figure 4C). Data on serum 25(OH)D for the 4 summer months (May to August) are absent as the adolescents were not sampled in this period due to school summer vacation. The serum 25(OH)D concentration of the remaining 8 months show a very steady pattern with monthly means around 40 nmol/L. These 8 months overlap directly with the 8 months of vitamin D winter in Tromsø during which UVB doses are too low to allow synthesis of pre-vitamin D_3_ in the skin. Also of note, the mean monthly serum 25(OH)D concentrations were, on average, 25 nmol/L lower in the adolescents compared to the adults over these 8 months in Tromsø.

## 4. Discussion

The present work provides modeled UVB doses for a range of European locations, ranging from the South to the North of Europe, averaged into monthly estimates but arising from daily data for a 10-year period (2003–2012). Importantly, the UVB doses in the present work were weighted with the vitamin D action spectrum. These new European data complement data on the distribution of solar UV radiation (also weighted according to the vitamin D action spectrum) over the USA and Canada [23]. The data shows a clear trend of decreasing UVB availability on moving from South to North within Europe, which would be predicted by the increasing SZA as one moves from ~35° N to ~69° N. There was an almost six-fold difference in mean yearly modeled UVB dose between these two latitude extremes within our selection of European locations. In general, within a latitude band, such as 50–60° N or 60–70° N, mean yearly modeled UVB availability was relatively similar across different European countries (only 1.1 to 1.2-fold difference). It is important to note, however, that these were, for the most part, comparisons of mean UVB doses for a country, and, as such, do not reflect within country differences, which can occur. In the case of Greece and Norway in the present analysis, there were clear differences in modeled UVB dose in the different locations within either country, which were separated by 2 to 9 degrees of latitude. However, even at the same latitude, differences can occur. For example, we have recently shown significant differences in UVB availability between inland and coastal areas at the same latitude within the UK, due to different cloud conditions [9]. Importantly, the present UVB doses across Europe were modeled in realistic weather conditions taking cloud coverage and optical depth into account. 

The impact of changing SZAs due to seasonality on UVB availability was also evident in every location examined in the present analysis. As the Earth orbits the sun the SZA changes, being at their smallest in summer (the sun is more overhead resulting in higher UVB levels) and largest in winter (sun is more to the horizon resulting in lower UVB levels) [3]. The magnitude of the maximum and minimum monthly mean UVB in summer and winter, respectively, differed by latitude as expected, with lowest maximum and deepest minimum UVB doses occurring at highest latitude (Tromsø, Northern Norway). The modeled UVB doses also highlight very clearly the increasing length of vitamin D winters within Europe as one moves from ~35° N (essentially no vitamin D winter) to ~69° N (eight months of vitamin D winter). Much of mid-Europe has a vitamin D winter which lasts from around four to six months, and this period of much diminished, or even absent, potential for synthesis of vitamin D in the skin undoubtedly contributes to the relatively high prevalence of vitamin D deficiency in adults during winter in this region (typically in the range 13% to 36% [2]). However, the UVB availability data from the present analysis also highlights very clearly how proactive interventions can overcome the environmentally-induced constraints in UVB availability during the vitamin D winter periods. For example, despite more abundant UVB availability in mid-Europe (45–60° N) compared to the more northerly regions (>60° N), population vitamin D status is generally better in these Northern European countries compared to those in mid-Europe [2,6,24,25]. 

In the present work, based on standardized serum 25(OH)D data which allows for more valid between-country examinations [7], we showed that adults in Iceland and Tromsø, Northern Norway had much higher serum 25(OH)D concentrations during winter compared to that in adults in Ireland. We postulate that the lower prevalence of vitamin D deficiency in northern European versus mid-latitude countries is due to a combination of factors including traditional behavior (especially amongst older adults, e.g., siesta in Mediterranean countries versus active middle of day at higher latitudes), paler skin pigmentation amongst the indigenous population at higher latitudes [26], more conscious sun-seeking behavior, clothing and holidays amongst the northern Europeans, but also, importantly, differences in vitamin D intake. For example, data from the most recent national nutrition survey shows that mean dietary intakes of vitamin D by Irish adults are relatively low (5.0 μg/day and 6.9 μg/day for 18–64 and ≥65 years, respectively [20,27]) and, as shown in the present analysis, are not sufficient to maintain serum 25(OH)D > 30 nmol/L in 17.8% of the population during the vitamin D winter for Ireland. In contrast, the prevalence of vitamin D deficiency during the respective vitamin D winters in adults from Iceland and Tromsø were noticeably lower (8.5% and 1.1%, respectively) compared to estimates from Ireland, and indeed other mid-European countries [2]. Unfortunately, neither the AGES-Reykjavik cohort nor the Tromsø (Cycle 6) study assessed vitamin D intakes which is a limitation for the present work, however, data from the Icelandic National Nutrition Survey suggest mean dietary intakes of vitamin D of 12.9 μg/day and 8.4 μg/day for men and women aged ≥ 65 years, respectively [28], while the Norkost survey of adults aged 18–70 years in Norway in 2011 suggest mean dietary intakes of vitamin D of ~12 μg/day and ~7 μg/day from all sources and food sources only, respectively [29]. These higher intakes of vitamin D in Iceland and Tromsø arise from the use of cod liver (in traditional foods [30]) and/or cod liver oil (as a supplement) and other vitamin D supplements together with higher consumption of fatty fish [31]. Older estimates of mean vitamin D intakes by adults in the fourth cycle of the Tromsø study were 7.1 μg/day and 6.5 μg/day for men and women, respectively [32], but these were from 1994–1995. The 1997 Norkost survey of adults aged 16–79 years in Norway report mean daily intake of 5.8 μg/day and 4.0 μg/day for men and women, respectively [33]. 

The higher vitamin D intakes in the Northern countries may facilitate serum 25(OH)D concentrations which exhibit less seasonal fluctuations. In this way, the amplitude of increase in prevalence in vitamin D deficiency in winter compared to summer is much dampened compared to that seen in mid-European countries which have considerable seasonal fluctuations in serum 25(OH)D. For example, the prevalence of vitamin D deficiency in winter and summer was 2.2% and 0.5%, respectively, in Tromsø, and 9.7% and 7.1%, respectively, in Reykjavik. In contrast, the prevalence of vitamin D deficiency in winter and summer was 23.8% and 0.8%, respectively, in Ireland. It is also possible that some of the protective effect on winter vitamin D status in these Northern countries relates to release of vitamin D from stores in subcutaneous adipose tissue and muscle [34,35]. These stores could be built up following UVB exposure during the preceding summer-time. Of note, summer peak serum 25(OH)D concentrations were higher in Tromsø than Ireland, despite 1500 Jm^−2^ lower mid-summer UVB doses in the former. Also other sun exposure characteristics, such as taking a sun holiday at more south latitudes during the preceding summer has been shown to be positively associated with vitamin D status in middle-aged women in Northern Norway [30]. Lastly, it is important to stress there may be other potential contributory factors for the difference is vitamin D status between Norway and Ireland. For example, the proportion of smokers in the population may be higher in Ireland than Norway based on lung cancer mortality data [36], and smoking has been associated with lower serum 25(OH)D concentrations [37]. 

The collective data on UVB availability as well as population serum 25(OH)D for adults and adolescents in Tromsø illustrate how dietary vitamin D input of sufficient amount can offset the limited UVB availability, and thus potential to synthesise vitamin D in the skin, during its vitamin D winter. Adolescents in Tromsø had, on average, 25 nmol/L lower mean monthly serum 25(OH)D concentration compared to that in adults over the same eight months of vitamin D winter and with the same UVB availability. While a number of factors may underpin these differences, one of the most likely is the difference in vitamin D intake arising from different dietary practises between adults and adolescents in Tromsø. Estimates of mean vitamin D intakes by Norwegian adolescents (aged 13 years) were 2.5 μg/day which excluded that coming from supplements [38]. However, these estimates would increase to ~7.5 μg/day if the 40% daily vitamin D supplement use, as reported in the most recent adult survey [28], were the case in the adolescents. However, daily use of cod liver oil in the Fit Futures study was reported as only 17% and a further 35% used it sometimes [18]. Other vitamin and mineral supplements were also used, 19% used daily and a further 44% used sometimes [18]. In relation to different dietary practises, it is interesting that a traditional fish liver dish called mølje, made mainly from cod liver and its consumption following the seasonal harvests for cod (mid-December until April), has been shown to be an important contributor to vitamin D intake in Northern Norway [30], but the Norwegian Food Control Authority over a decade ago recommended that children, as well as pregnant women and women of child-bearing age, not to eat fish liver due to risk of persistent organic pollutants above tolerable levels [39]. The differences in vitamin D intake between adults and adolescents may also relate to other differences in dietary patterns beyond fish, e.g., in meat and eggs, which have been shown to be important sources of not only vitamin D per se, but also 25(OH)D [40]. 

## 5. Conclusions

In conclusion, modeled UVB dose data for European countries highlight differences in vitamin D effective UVB availability which occur due to latitude and seasonality. They provide additional insight into vitamin D status within the population, illustrating the limits to potential for cutaneous synthesis of the vitamin. This work shows that UVB availability alone cannot explain a population’s vitamin D status, and we suggest that UVB availability and diet, as well as other lifestyle factors, must be assessed in seeking solutions to vitamin D deficiency. 

## Figures and Tables

**Table 1:** Modeled mean yearly ultraviolet B (UVB) doses, duration of vitamin D winter, proportion of a typical year with UVB doses less than 1000 Jm^−2^ and cosine of solar zenith angle over a 10 year period (2003–2012) at selected European countries and regions.

Country/Region	Latitude (° N)	Coordinates for Modeling	Modeled Yearly UVB (Jm^−2^) *		Vitamin D Winter ** (months)	% of the Year <1000 Jm^−2^	Cosine of Solar Zenith Angle ***
			Mean	SD			
Greece:							
Crete	35	35.2° N, 24.9° E	5500	3600	0	7	0.95
Athens	37	38.0° N, 23.7° E	4800	3400	2	16	0.94
Thessaloniki	40	40.6° N, 22.9° E	4100	3000	2	21	0.93
Germany	47–55	Grid 1: 47.4 to 49.9° N, 6.4° E to 13.8° E; Grid 2: 50.0 to 51.9° N, 6°0′ E to 15°0′ E; Grid 3: 52.0 to 54.4° N, 7°0′ E to 14.7° E	2500	2100	4	40	0.87
Ireland	51–54	51.4–54.5° N,5.4–10.5° W	2100	1900	5	43	0.85
UK	50–59	50.5–58.0° N,4°5′ W–1°2′ E	2000	1800	6	43	0.83
Netherlands	52	52.3° N to 52.6° N, 4.9°–5.1° E	2200	2000	5	42	0.86
Denmark:							
Copenhagen	56	54°45′ N to 55°51′ N, 11° 51′ E to 12°30′ E	2000	1900	6	45	0.83
Aarhus	56	56.2° N, 10.2° E	1900	1800	6	48	0.82
Finland	60–70	Grid 1: 59.8° to 63.8° N, 21.2° E to 30.5° E Grid 2: 63.9° N to 66.7° N, 23.7° E to 30°0′ E.	1400	1600	6	55	0.75
Iceland, Reykjavik	64	64° 09′ N, 21° 57′ W	1200	1400	7	60	0.76
Norway:							
Oslo	60	59.9° N, 10.7° E	1700	1700	6	51	0.79
Tromsø	69	69.4° N, 18.6° E	900	1200	8	64	0.68

* Rounded to the nearest 100 Jm^−2^. ** Number of months with mean monthly UVB doses below 1000 Jm^−2^. *** Estimated at true solar noon on 21 June in 2008.
